# Editorial: Mental health and sequels to violence in primary health care

**DOI:** 10.3389/fpubh.2024.1423765

**Published:** 2024-05-16

**Authors:** Thomas Wenzel, Jan Ilhan Kizilhan

**Affiliations:** ^1^Department Psychiatry and Psychotherapy, Medical University of Vienna, Vienna, Austria; ^2^World Psychiatric Association Scientific Section on Psychological Aspects of Persecution and Torture, Geneva, Switzerland; ^3^University of Duhok, Duhok, Iraq; ^4^Institute for Transcultural Health Science, State University Baden-Württemberg, Villingen-Schwenningen, Germany

**Keywords:** violence, gender, health care professionals, primary care, refugees, trauma, PTSD, primary health care

Violence is a critical causative factor in mental health problems that is, in the global context, rather increasing (1) despite multiple conventions and strategies aiming at reducing its prevalence. Physical violence is usually combined with psychological violence, and psychological forms of aggression and violence are probably even more common in many settings and countries, ranging from discrimination and persecution to domestic violence (2–6). Primary health care institutions are frequently a point of first contact for victims, even if victims often might hesitate to share their experience, while their physical injuries or psychological symptoms might be strong indicators of violence encountered. Health care experts are in consequence obliged to recognize, document, support and follow up on suspect injuries, if necessary also referring cases for further investigation to legal authorities. Mental health sequels can include a wide range of symptoms, including, but by no means limited to those of posttraumatic stress disorder, complex posttraumatic stress disorder, culture based idioms of distress or more unspecific symptoms such as those of depression or somatoform disorders. They can be long lasting, cause substantial suffering or even impairment, and might if not recognized or untreated lead to secondary problems such as family conflicts, suicide, or substance abuse to cope with psychological sequels. A comprehensive and well considered strategic approach is required to better understand and address this often challenging situation, and address the diverse situations and needs of different victim groups in a way considering their respective situations and cannot be limited to Psychiatric interventions, as also proposed by several authors in our Research Topic.

Migrants and refugees, who most frequently have encountered different forms of violence with often restricted and unequal access to the health care system are an issue of special concern in this context. Kienzler of the Kings College in London has explored the impact of systemic injustices and social inequalities, that could be seen as forms of the “structural” variation of violence on a group of refugees in the United Kingdom, a country where basic human rights have recently been under continuous attack with the risk of a grave negative impact on mental health especially in vulnerable groups. Kienzler is using qualitative research, that is best fitted to provide for a better understanding of newly identified problems and proposes possible solutions to avoid factors leading to increased suffering in refugee and migrant groups.

In this Research Topic, several authors from the People‘s Republic of China further have explored in turn the experience of violence encountered by care givers and health professionals as often neglected further group of victims [see also (7)]. Xu et al. have explored the “vertical” violence in an especially vulnerable group of health care professionals, i.e., nursing interns, using an Importance-Performance Analysis (IPA) approach. Results underline the responsibility of institutional managements to recognize and address this problem and create supportive environments for their important work.

Chen et al. have conducted a survey with a large sample to explore the continuity of instances of violence experienced by doctors in the PRC in spite of government efforts to reduce the frequency of such events. The authors give an overview of the present health care system and report, that violence has decreased, but is still a common problem for doctors who are often targeted by, mostly non-physical, violence, on levels similar to other countries such as the US. The study results might encourage further research to better understand the reasons for the different forms of violence encountered by health care professionals, not only in China, and address their backgrounds to better guide interventions including, but not limited to legal strategies. Wen et al. have explored the situation of health care professionals at risk for burn-out that is, as also observed by other researchers, aggravated by the COVID pandemic crises. Balancing family needs, as a basic human need, and workload might be a special challenge and might lead to a loss of experienced professionals in their work places. Management should recognize and address this problem, again not only in China.

Yan et al. identified and explored a further critical but so far largely neglected area of concern in the context of violence, that is the feeling of safety and protection of inpatients in hospitals. The authors used a meta-analytical approach based on published qualitative studies identified in online databases, using an innovative approach of second-level analysis and identified a number of relevant issues, focusing on relevant key factors, identified as dignity, responsibility, stability and control of the hospital environment.

The studies included in this Research Topic demonstrate that violence in different forms including also structural violence and the neglect of the needs of helpers, so far insufficiently covered in prior research, should be considered by future projects and interventions in this area. Vulnerable groups such as refugees as direct victims should receive better support and protection, again considering the neglect of human rights also in “first world” countries such as the UK.

## Author contributions

TW: Writing – original draft, Writing – review & editing. JK: Resources, Writing – review & editing.
